# Quantifying domain-specific relevance of computational biology Wikipedia articles using TF-IDF and cosine similarity

**DOI:** 10.1093/bioinformatics/btag278

**Published:** 2026-07-07

**Authors:** Arya Pradeep Menon, Trenton Davis, Megha Hegde, Nicolas C Näpflin, Audra Anjum, Sarah Barman, Dan DeBlasio, Pradeep Eranti, Jean-Christophe Nebel, Nelly Sélem-Mojica, Sayane Shome, Lonnie R Welch, Alastair M Kilpatrick, Farzana Rahman

**Affiliations:** School of Computing and Mathematics, Faculty of Engineering, Computing, and Environment, Kingston University, London, KT1 2EE, United Kingdom; School of Electrical Engineering and Computer Science, Ohio University, Athens, OH 45701, United States; School of Computing and Mathematics, Faculty of Engineering, Computing, and Environment, Kingston University, London, KT1 2EE, United Kingdom; Department of Molecular Life Sciences and Swiss Institute of Bioinformatics, University of Zurich, Zurich 8057, Switzerland; Office of Instructional Design, Ohio University, Athens, OH 45701, United States; School of Computing and Mathematics, Faculty of Engineering, Computing, and Environment, Kingston University, London, KT1 2EE, United Kingdom; Ray and Stephanie Lane Computational Biology Department, Carnegie Mellon University, Pittsburgh, PA 15213, United States; Université Paris Cité, Inserm, T3S, Paris 75006, France; School of Computing and Mathematics, Faculty of Engineering, Computing, and Environment, Kingston University, London, KT1 2EE, United Kingdom; Centro de Ciencias Matemáticas, Universidad Nacional Autónoma de México, Morelia 58089, México; School of Medicine, Stanford University, Stanford, CA 94305, United States; School of Electrical Engineering and Computer Science, Ohio University, Athens, OH 45701, United States; Centre for Regenerative Medicine, Institute for Regeneration and Repair, The University of Edinburgh, Edinburgh, EH16 4UU, United Kingdom; School of Computing and Mathematics, Faculty of Engineering, Computing, and Environment, Kingston University, London, KT1 2EE, United Kingdom

## Abstract

**Motivation:**

Wikipedia is one of the world’s most visited websites and serves as the principal open educational resource for computational biology. However, identifying which articles are most relevant to distinct sub-disciplines of computational biology remains largely subjective.

**Results:**

This study collected short descriptions for 22 Communities of Special Interest (COSI) groups maintained by the International Society for Computational Biology and downloaded 1536 computational biology articles from English Wikipedia. Following standard text preprocessing, COSI descriptions and Wikipedia articles were embedded in a common TF-IDF vector space. Semantic relatedness was quantified using cosine similarity, yielding a real-valued relevance matrix that maps each COSI to the most pertinent computational biology articles. The resulting scores, typically low in absolute value, captured nuanced differences: general-interest pages such as ‘Computational biology’ and ‘Bioinformatics’ ranked highest, whereas niche pages showed high relevance only for specific COSIs. Unsupervised analysis using principal component analysis, *k*-nearest neighbours, and Leiden community detection revealed clusters of articles corresponding to the particular COSIs and highlighted inter-COSI relationships. This automated pipeline reduces bias compared with manual tagging and enables more precise curation of domain-specific educational resources.

**Availability and implementation:**

The relevance matrix developed in this study is available in the Zenodo repository (doi: 10.5281/zenodo.18311878).

## 1 Introduction

The online encyclopaedia Wikipedia is regarded as one of the most important channels for the public communication of science (Jemielniak and Aibar 2016, Kilpatrick *et al.* 2022) and is the most frequently accessed educational resource in computational biology, with the most popular pages being viewed hundreds, and often thousands, of times daily.

The Computational Biology taskforce of WikiProject Molecular Biology (formerly WikiProject Computational Biology) is an international community of Wikipedia editors, formed in 2007 to organize and improve Wikipedia articles relating to aspects of computational biology and bioinformatics (O’Neill *et al.* 2017). Many of these editors are academic or industrial scientists with some expertise in computational biology, who provide important links between their particular specialism, Wikipedia and any professional organizations of which they are a member. The taskforce now oversees around 1500 articles relating to ‘classical’ bioinformatics (e.g. sequence analysis, genomics and proteomics), modelling of biological systems, bioinformatics data types and resources, algorithms pertinent to computational biology, biographies of notable computational biologists and relevant institutions of global prominence.

Since 2014, the International Society for Computational Biology (ISCB) has established Communities of Special Interest (COSIs) (https://www.iscb.org/about-iscb/cosis) as self-organized, topic-focused subcommunities within computational biology. COSIs facilitate ongoing scientific interaction throughout the year, rather than solely convening during the ISCB’s annual conference tracks. Each COSI centers on a distinct area of computational biology and bioinformatics, providing a dedicated forum where researchers collaborate on domain-specific research questions and opportunities. These communities communicate through regular meetings and online platforms, enabling continuous engagement and knowledge exchange in a vibrant virtual environment. This year-round model of engagement sustains active collaborations and discourse outside of traditional conferences. As of 2020, ISCB has 22 such COSIs, each representing a major sub-discipline of computational biology and bioinformatics. Collectively, the COSIs span a broad range of topics; e.g. from structural bioinformatics and data visualization to regulatory genomics and machine learning in systems biology, reflecting the diversity of the computational biology field.

Both the ISCB and the Wikimedia Foundation, the non-profit organization which operates Wikipedia, share a common mission of promoting open access to scientific information and educational resources. To this end, ISCB conferences have previously hosted several Wikipedia-based tutorials and editathons (O’Neill *et al.* 2017), and the ISCB also runs a long-standing Wikipedia editing competition for students and postdocs (Bateman *et al.* 2013, Kilpatrick 2016, Kilpatrick *et al.* 2026).

There are benefits to both parties in identifying Wikipedia articles that are topically relevant to specific COSIs(Kilpatrick *et al.* 2022). Formalizing lists of topically relevant articles would allow COSIs to point to open educational resources that fall within the disciplinary scope of their domain. Encouraging COSI members to improve Wikipedia articles in which they are experts would also improve the representation of computational biology online; for academics, improving relevant Wikipedia articles represents a professional responsibility (Callis *et al.* 2009) and effective communication of bioinformatics to a range of audiences has been identified by the ISCB as a core competency for those in bioinformatics roles (Brooksbank *et al.* 2024). Wikipedia would also benefit, through gaining a new community of highly skilled editors who have some personal investment in ensuring topics pertaining to their (sub)field are both visible and well-written.

Our team previously developed and published a COSI-Article matrix (Kilpatrick *et al.* 2022), which linked COSIs to relevant Wikipedia articles, thereby defining domain-specific educational resources for computational biology and facilitating COSI members to improve Wikipedia articles in which they are experts. However, this resource still has several limitations. Firstly, the assessment of *topical relevance*, or whether the subject of a Wikipedia article aligns with or falls within the disciplinary scope of a COSI, is inherently subject to unconscious bias and human subjectivity, reflecting differences in participants’ backgrounds and levels of domain-specific expertise. Secondly, the COSI–Article matrix captures article–COSI relationships using a binary relevant/not-relevant scheme, which does not reflect varying degrees of relevance. Introducing a relevancy scale that uses a real-number score would provide finer-grained information about article relevance, which is almost certainly different across COSIs.

The rapid expansion of generative AI and text-mining models suggests an automated method for scoring the relevance of Wikipedia articles to given ISCB COSIs, which would reduce the human biases identified in the previous COSI-Article matrix. Methods such as keyword matching (for instance, via Term Frequency-Inverse Document Frequency; TF-IDF) (Salton and Buckley 1988), can be used to generate a real-valued matrix of relevancy scores, given Wikipedia article text and COSI keywords or descriptions.

In this study, we develop a text analysis pipeline which extracts text from Wikipedia articles and applies TF-IDF to determine relevancy for each of the ISCB COSIs. We apply unsupervised learning to the resulting data, which gives valuable insights into computational biology representation on Wikipedia at a domain-specific level.

## 2 Materials and methods

### 2.1 Data collection

Short textual descriptions for each COSI were downloaded from the ISCB website (https://www.iscb.org/about-iscb/cosis) in December 2025. Text from Wikipedia articles relating to computational biology (*n* = 1536) was retrieved via the English Wikipedia MediaWiki Action API endpoint (https://en.wikipedia.org/w/api.php) using the requests Python library in December 2025. Briefly, the API was queried to return the names for all articles tagged by Wikipedia users as being in the category ‘Computational Biology articles’. Technical pages such as category pages, Wikipedia templates and discussion pages were filtered from the list, as were redirect pages. For each article, the Wikipedia API was queried to return the text content, which was filtered to remove section headings, reference text and any other Wikipedia tags.

### 2.2 Text preprocessing

**Figure 1:**
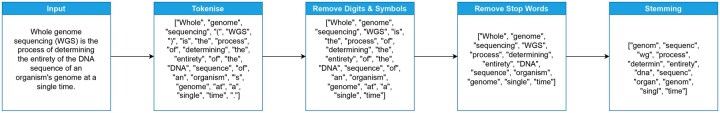
Text preprocessing workflow for COSI descriptions and Wikipedia articles.

COSI descriptions and Wikipedia articles were subject to systematic preprocessing, including tokenization, removal of irrelevant data and stemming (Fig. 1). Tokenization divided the text into discrete tokens such as individual words and punctuation marks, facilitating downstream processing. The tokenized output was then filtered to exclude digits, symbols, and ‘stop words’. Stop words are defined as high-frequency words that contribute little information to topic discrimination (e.g. ‘is’, ‘to’, and ‘or’) (Sarica and Luo 2021). By removing non-alphanumeric characters and digits, the focus on semantic content directly relevant to COSIs was maintained. The stemming process reduced words to their root form, ensuring consistent representation (e.g. ‘sequenc’ instead of ‘sequence’ or ‘sequencing’).

### 2.3 Feature extraction

The preprocessed data was represented as unigrams (individual words), for both COSI descriptions and Wikipedia articles. A TF-IDF matrix was calculated to assess the importance of each word in relation to a document collection. The TF component measures how frequently a word appears in a document [Equation (1)]. The IDF component measures how rare a word is across the corpus [Equation (2)]. The resulting product [Equation (3)] represents the TF-IDF score.

TF-IDF quantifies the significance of each term, distinguishing common but less informative words from rarer and highly informative terms, yielding a robust feature representation for similarity analysis.


(1) $$TF=\frac{\text{Number}\;\text{of}\;\text{times}\;a\;\text{term}\;\text{appears}\;\text{in}\;a\;\text{document}}{\text{Total}\;\text{number}\;\text{of}\;\text{terms}\;\text{in}\;\text{the}\;\text{document}}$$

(2) $$\mathrm{IDF}=\log\left(\frac{\text{Total}\;\text{number}\;\text{of}\;\text{documents}}{\text{Number}\;\text{of}\;\text{documents}\;\text{containing}\;\text{the}\;\text{term}}\right)$$

(3) TF-IDF=TF×IDF

### 2.4 Cosine similarity

Cosine similarity was used to quantify the topical relevance of each article to each COSI. The cosine similarity score enables standardized comparisons of article relevance, independent of article length. A higher score indicates a stronger relevance with a given COSI. Equation (4) defines the calculation of cosine similarity, where *A* and *B* represent the TF-IDF weighted unigram vectors of a COSI and an article, respectively. Each vector dimension corresponds to a unigram, and its value represents the TF-IDF weight of that unigram in the document.


(4) $$\text{Cosine}\;\text{Similarity}(A,B)=\frac{A\cdot B}{\left|A|\times |B\right|},$$

where $A=[a_{1},a_{2},\ldots ,a_{n}]$, $B=[b_{1},b_{2},\ldots ,b_{n}]$ with $a_{i}$ and $b_{i}$ denoting the TF-IDF weight of the *i*-th unigram in the COSI and article respectively and the magnitudes |*A*| and |*B*| are calculated as:


(5) $$|A|=\sqrt{a_{1}^{2}+a_{2}^{2}+\ldots +a_{n}^{2}}\;\text{and}\;|B|=\sqrt{b_{1}^{2}+b_{2}^{2}+\ldots +b_{n}^{2}}.$$

Tokenization was performed with the Natural Language Toolkit (NLTK) Python package (Bird and Loper 2004). TF-IDF and cosine similarity were computed with the Scikit-learn and NumPy Python packages (Pedregosa *et al.* 2011, Harris *et al.* 2020).

### 2.5 Unsupervised analysis and clustering

**Figure 2:**
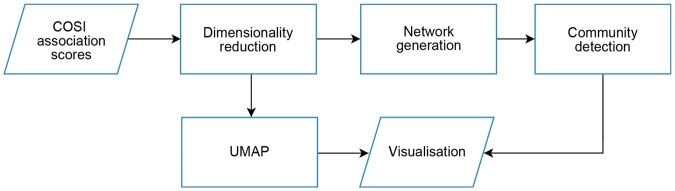
Unsupervised learning pipeline to identify clusters of Wikipedia articles based on COSI association scores.

We developed a pipeline to perform unsupervised analysis and clustering of data from the TF-IDF analysis. Initial dimensionality reduction via Principal Component Analysis (PCA) was performed on the data to identify the components that capture the most variation within the data. The elbow method was used to identify the optimal number of principal components (PCs) for downstream analysis. Next, a network was generated using a combination of *k*-Nearest Neighbours and Shared Nearest Neighbours (SNN). The network connects articles that are similar and removes connections between those that are dissimilar, based on Euclidean distance. In constructing the graph, a similarity threshold that determines whether an edge is drawn is chosen as the lowest value that maintains overall graph connectivity while still preserving meaningful distinctions between COSIs or articles. Leiden community detection was performed on the SNN graph to identify groups of articles that are like one another. Finally, Uniform Manifold Approximation and Projection (UMAP) was generated using the PCs identified previously. Community labels and COSI relevance scores were overlaid on the UMAP separately to visualize the results, as appropriate. The complete unsupervised analysis and clustering workflow is presented in Fig. 2.

## 3 Results

### 3.1 TF-IDF generates a COSI-Article relevancy matrix

To explore if the TF-IDF method was sensitive to the specific subfields of each COSI, we extracted word contribution scores for each COSI as computed by TF-IDF and aggregated them across all Wikipedia articles. The top-scoring words for selected COSIs are presented in Table 1. We found TF-IDF often identified relevant and specific keywords for each COSI. For example, ‘structur’ (0.366) was the highest scoring word for the 3DSIG COSI, which focuses on structural bioinformatics and computational biophysics. Similarly, ‘translat’ (0.180) and ‘medicin’ (0.133) scored highly for the TransMed (translational medicine informatics and applications) COSI. However, we also note the appearance of general words such as ‘biolog’, ‘scienc’, and ‘data’ which also ranked highly for many COSIs.

**Table 1:** Word roots with the highest contribution scores for selected ISCB COSIs.

3DSIG	Education	HiTSeq	RegSys	TransMed
structur (0.366)	biolog (0.060)	sequenc (1.244)	genom (0.530)	translat (0.180)
protein (0.181)	scienc (0.048)	analysi (0.159)	gene (0.197)	medicin (0.133)
cell (0.121)	program (0.042)	algorithm (0.151)	studi (0.138)	data (0.084)
biolog (0.119)	life (0.041)	data (0.146)	ismb (0.070)	profil (0.067)
model (0.102)	bioinformat (0.040)	biolog (0.067)	organ (0.057)	approach (0.041)
molecular (0.099)	support (0.039)	includ (0.060)	system (0.053)	scientist (0.036)
includ (0.086)	develop (0.029)	method (0.058)	confer (0.045)	involv (0.033)
process (0.070)	major (0.028)	transform (0.047)	meet (0.027)	consist (0.033)
design (0.070)	educ (0.028)	present (0.043)	method (0.027)	clinic (0.032)
function (0.052)	share (0.028)	multipl (0.036)	activ (0.025)	analysi (0.031)

We similarly extracted TF-IDF values for the twelve Wikipedia articles assessed as being relevant to computational biology and in Wikipedia’s peer-reviewed ‘Good Article’ (‘useful to nearly all readers, with no obvious problems; approaching the quality of a professional publication’) article quality category. The top-scoring words for selected articles are presented in Table 2. We found that, for these articles, TF-IDF was able to identify specific and relevant words which could be used in computing relevancy scores for each COSI; in contrast to COSI-level scoring, we found fewer highly ranked general terms.

**Table 2:** Word roots with the highest contribution scores for selected Good Articles relating to computational biology.

Circular permutation in proteins	Combined DNA index system	Genome-wide association study	Intelligent systems for molecular biology	Transcriptomics technologies
permut (0.724)	codi (0.556)	gwa (0.609)	ismb (0.611)	transcript (0.380)
protein (0.392)	dna (0.273)	studi (0.428)	confer (0.535)	transcriptom (0.369)
circular (0.302)	profil (0.253)	snp (0.270)	held (0.295)	sequenc (0.297)
circularli (0.241)	crime (0.229)	genotyp (0.209)	meet (0.203)	gene (0.252)
saposin (0.121)	databas (0.220)	diseas (0.188)	iscb (0.153)	rna (0.200)
duplic (0.110)	loci (0.197)	allel (0.147)	keynot (0.115)	read (0.192)
fusion (0.101)	convict (0.180)	variant (0.134)	track (0.106)	express (0.169)
fission (0.084)	nation (0.152)	odd (0.124)	present (0.099)	microarray (0.154)
polypeptid (0.072)	offend (0.147)	associ (0.117)	deleg (0.089)	array (0.151)
termini (0.072)	collect (0.132)	trait (0.108)	comput (0.079)	align (0.137)

**Figure 3:**
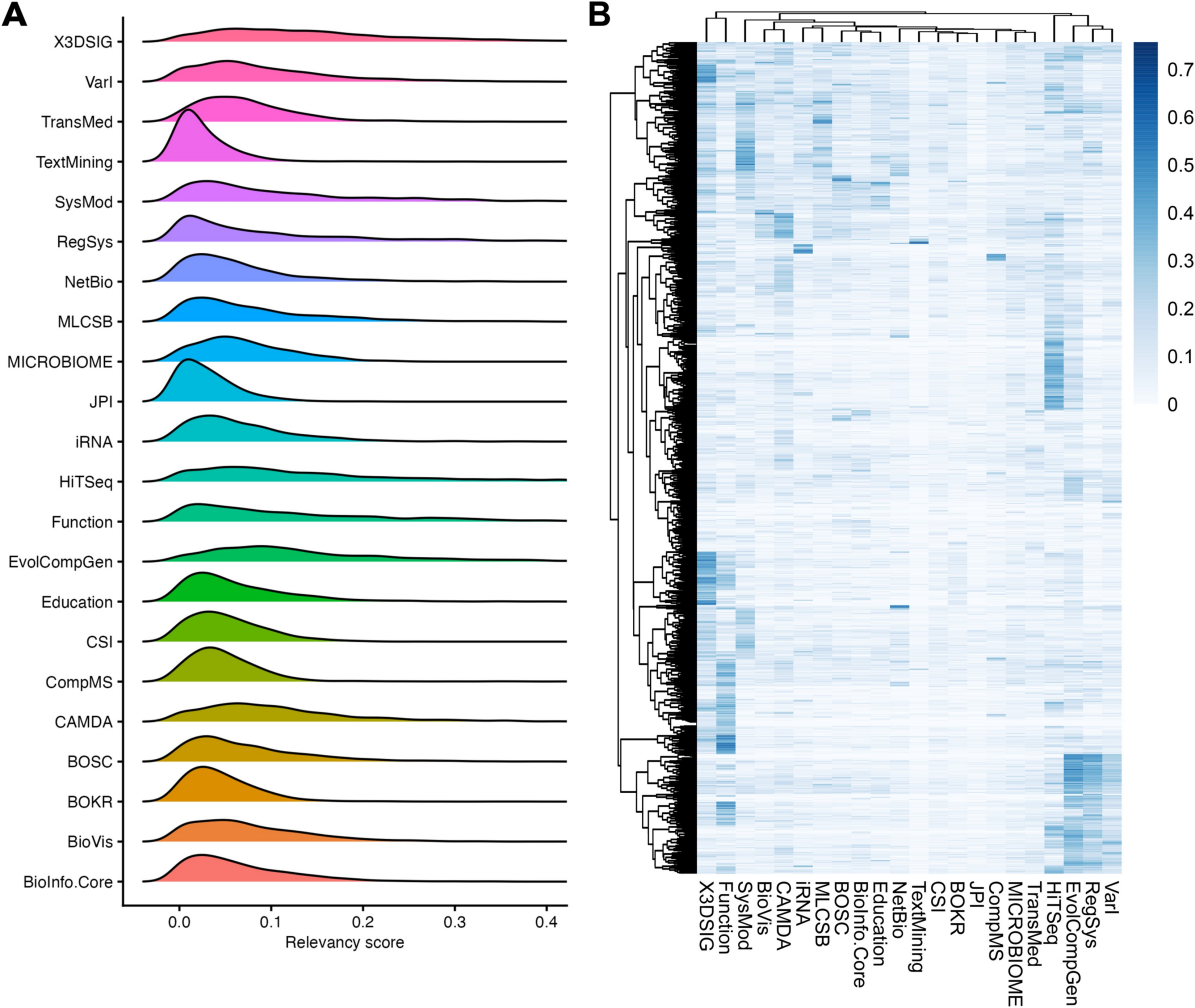
(A) Ridgeplot of Wikipedia article relevancy score distributions, split by COSI. The *x*-axis is limited to 0.4 for clarity. (B) Heatmap of relevancy scores, clustered by article and COSI.

The final output is a matrix of relevancy scores (ranging between 0 and 1), between Wikipedia articles (*n*=1536) and ISCB COSIs (n=22). Figure 3A presents relevancy score distributions, split by COSI. The absolute magnitude of the relevancy scores should be interpreted with caution. TF-IDF vectors are high-dimensional and sparse; therefore, even documents with related content often exhibit relatively low scores. Although mean relevancy for each COSI is relatively low (≤0.15), we note tighter distributions for the TextMining (mean 0.028) and JPI (0.031) COSIs, indicating generally low relevance to these COSIs across all articles, while COSIs such as EvolCompGen and HiTSeq have a wider distribution of relevancy scores.

Perhaps unsurprisingly, the article with the highest mean relevance was Computational Biology (0.214), with Systems Biology (0.176), Bioinformatics (0.175), and Biological Database (0.171) also ranking highly; ranking based on median relevance yielded very similar results. This data may be used to define article ‘importance’, or relevance to WikiProject Computational Biology, which is currently assessed by Wikipedia editors and therefore has an element of subjectivity. The data may also be used to prioritize articles for improvement, e.g. as of January 2026, the Computational Biology article is rated ‘C class’ on Wikipedia’s content assessment scale, indicating it ‘would not provide a complete picture for even a moderately detailed study’. Articles with the lowest mean relevance may require confirmation of their relevance to computational biology. However, those articles (which may have median relevance of 0 across all COSIs) may be highly specific to individual COSIs; e.g. YeTFaSCo, a short article about the Yeast Transcription Factor Specificity Compendium database, had a mean relevance score of 0.007, but was scored in the top 5% of articles for the CSI COSI (0.147).

Clustering articles by relevancy score (Fig. 3B) revealed a group of articles relevant to the VarI, RegSys, and EvolCompGen COSIs, and smaller groups of articles specific to single COSIs. As suggested by the distribution of relevancy scores, the TextMining and JPI COSIs were characterized by low relevance across almost all articles.

### 3.2 Unsupervised analysis reveals clustering of related articles

**Figure 4:**
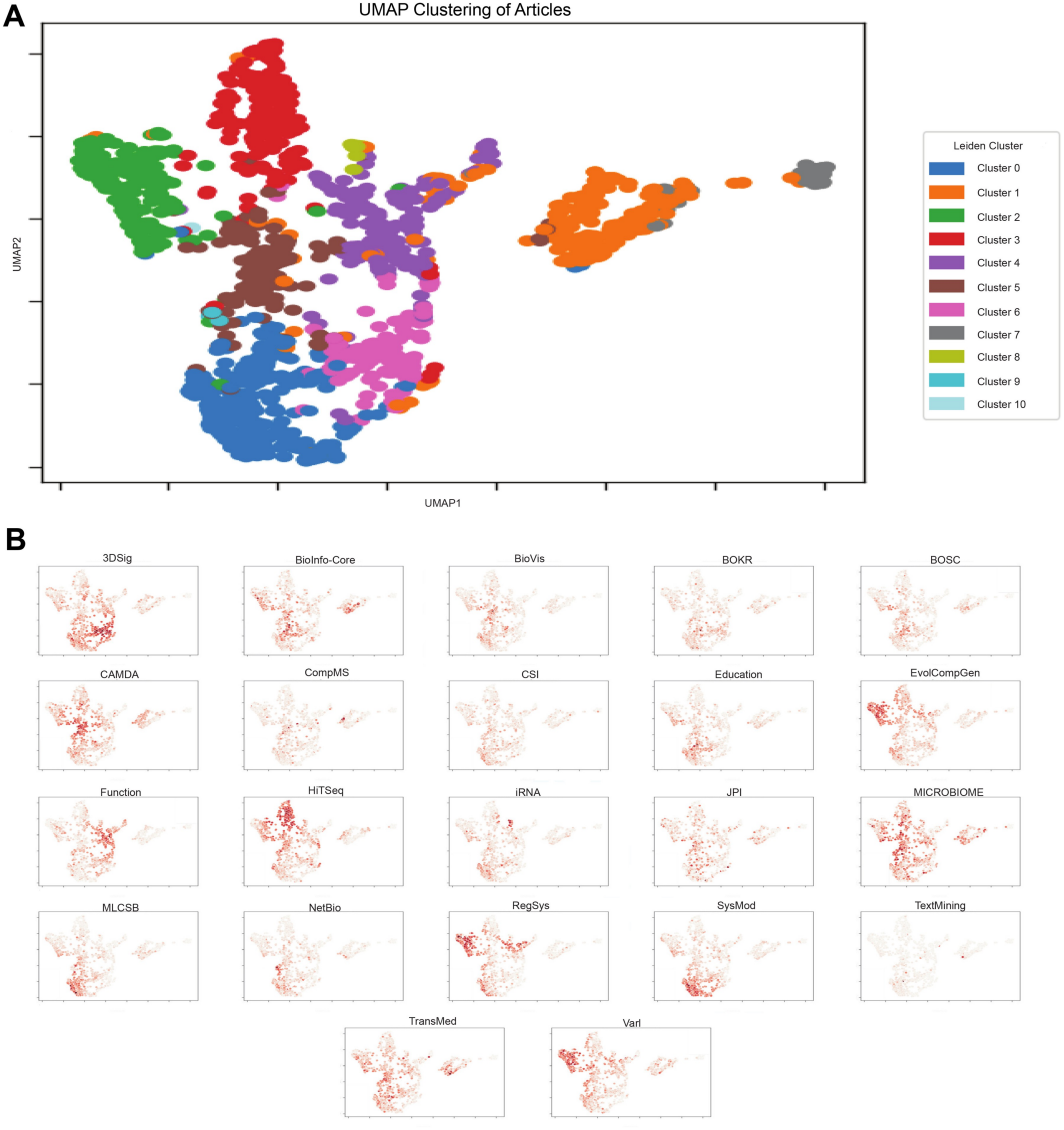
(A) Clustering of Wikipedia articles in UMAP space; each point represents a single article. (B) Articles visualized in UMAP space; colour represents relevancy score for each COSI.

To explore the data in the relevancy matrix further, we first analysed links between Wikipedia articles by performing dimensionality reduction on the data using Principal Component Analysis (PCA), and projecting the data into the lower-dimensional space with Uniform Manifold Approximation and Projection (UMAP). In UMAP space, articles with similar patterns of relevancy with respect to the COSIs appear in similar parts of the space. Unsupervised clustering of the data with *k*-nearest neighbours (see Section 2) revealed 11 distinct clusters of articles (Fig. 4A). The largest clusters in this analysis were robust to different choices of clustering resolution. Projection into UMAP space allowed us to visualize the data with respect to specific COSIs, colouring each article for relevance to a given COSI (Fig. 4B). For some COSIs, such as 3DSIG (Cluster 6) and HiTSeq (High Throughput Sequencing Algorithms & Applications; Cluster 3), the most relevant articles aligned well with previously identified clusters, and clusters were specific to these COSIs. Other identified clusters contained articles that were most relevant to several COSIs, e.g. Cluster 4 contained articles relevant to both Function (Gene and Protein Function Annotation) and RegSys (Regulatory and Systems Genomics) COSIs. Identified links between clusters and COSIs are presented in Table 3. Other COSIs such as TextMining (Text Mining for Healthcare and Biology) and CSI (Computational Systems Immunology) were not strongly associated with any identified cluster (Fig. 4B). Clusters 7, 8, 9, and 10 were smaller and did not have any strong overlap with any particular COSI.

**Table 3:** COSIs associated with selected clusters identified via the unsupervised learning pipeline.

Cluster 0	Cluster 1	Cluster 2	Cluster 3	Cluster 4	Cluster 5	Cluster 6
MLC SB	TransMed	EvolCompGen	HiTSeq	Function	CAMDA	3DSig
SysMod	BioInfo-Core	RegSys		RegSys	HiTSeq	
NetBio	CAMDA	VarI			MICROBIOME	
RegSys	MICROBIOME					

### 3.3 TF-IDF and unsupervised clustering reveals COSI relationships

**Figure 5:**
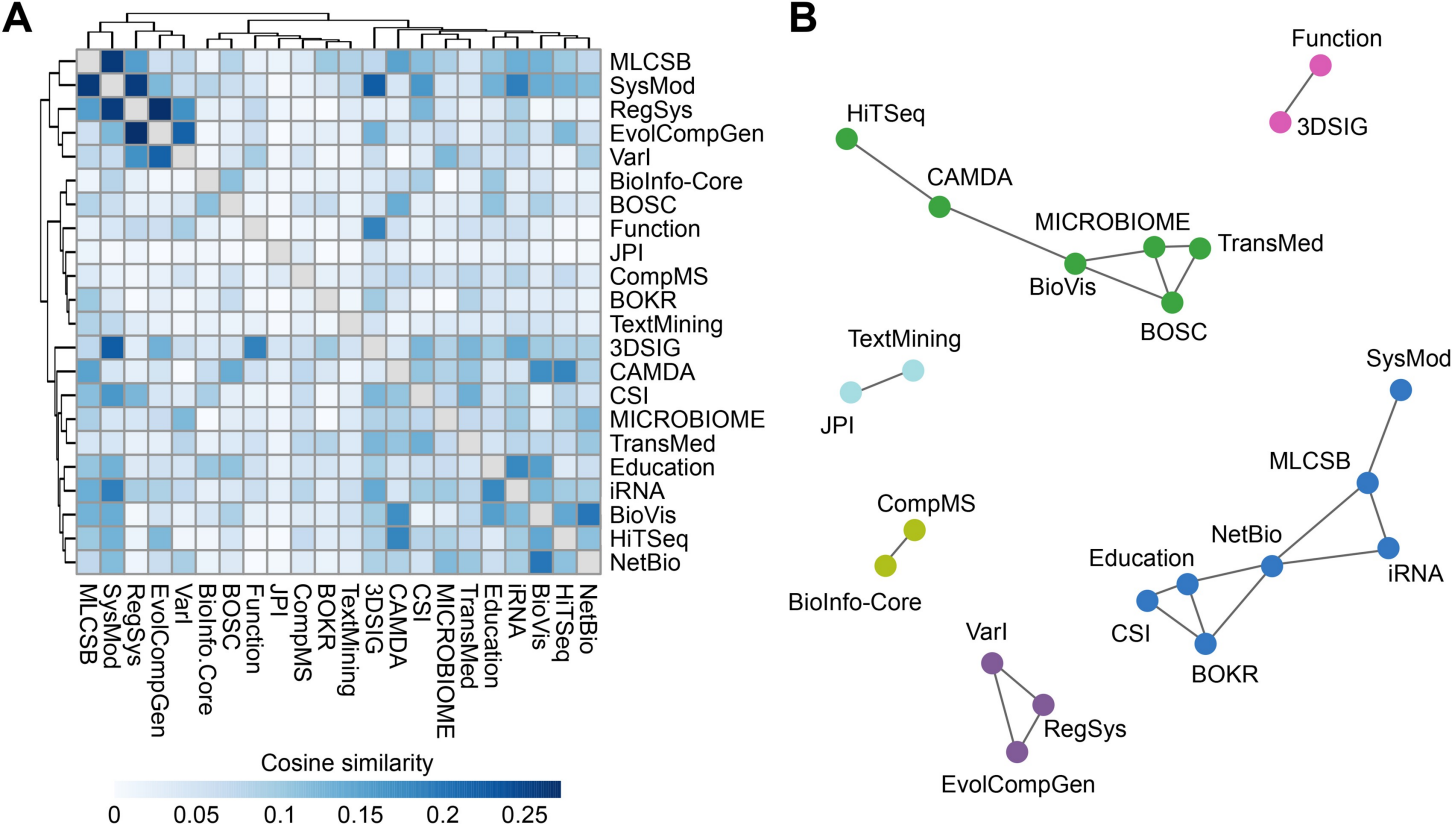
(A) Heatmap of pairwise cosine similarities between COSIs. (B) Unsupervised Leiden clustering of COSIs, based on relevancy matrix data.

To investigate potential links between COSIs, we computed cosine similarity scores using TF-IDF to compute the scores between each pair of COSIs and subject the resulting scores to hierarchical clustering (Fig. 5A). We also performed unsupervised dimensionality reduction and clustering of the relevancy matrix, based on COSIs rather than articles as above (Fig. 5B).

Cosine similarity identifies the strongest similarity (0.260) between the MLCSB (Machine Learning in Computational and Systems Biology) and SysMod (Computational Modelling of Biological Systems) COSIs, as part of a cluster of five related COSIs. MLCSB and SysMod COSIs also have moderately high pairwise cosine similarities with several other COSIs in other clusters, with SysMod and 3DSIG (0.225) being notably high. Similar to our previous observations (Kilpatrick *et al.* 2022), the JPI (Junior Principal Investigators) COSI was especially notable for its low cosine similarity (max = 0.045) with other COSIs.

When clustering the COSIs with the previously described unsupervised learning pipeline, the resulting structure closely mirrors the patterns obtained using direct cosine similarity. SysMod and MLCSB consistently group together, while RegSys, EvolCompGen, and Varl form a separate cluster using the unsupervised learning method. The COSIs CAMDA, HiTSeq, BioVis, TransMed, and MICROBIOME also cluster together under both approaches. However, with the unsupervised learning method, CSI, Education, NetBio, and iRNA join the SysMod-MLCSB group, which aligns with their relatively high similarity scores under cosine similarity. Function and 3DSIG likewise cluster together in both analyses.

## 4 Discussion

Wikipedia is the most widely accessed open educational resource in computational biology (Kilpatrick *et al.* 2022). In an educational context, the collaborative and editable nature of Wikipedia means that it has several potential advantages over standard textbooks. As noted above, generating a list of Wikipedia articles topically relevant to each ISCB Community of Special Interest (COSI) will be helpful in coordinating targeted editing and improvement efforts, including structured campaigns or editathons, and in supporting the creation of curated sub-domain primers as open educational resources for students and newcomers in Wikipedia editing. Additionally it will ensure the deliberate allocation of editor stewardship across articles relevant to specific COSIs, promoting sustained topical expertise and quality. Over time, continued use of the matrix will reveal trends and shifts in the sub-domain’s focus, providing insight into its evolution.

Adhering with the ISCB’s mission, each COSI is characterized by a distinct set of research themes, methodological approaches, and educational priorities. The textual or terminological descriptions associated with COSIs and their associated research articles are therefore not merely organizational summaries, but concise representations of the conceptual but often terminological boundaries that delineate subfields within the discipline. This makes COSIs a suitable reference framework for investigating domain specificity in computational biology educational resources.

Although the ISCB COSIs were formed organically, with evolving and community-defined thematic boundaries, many of the latent topical structures underlying these communities can be recovered using modern text analysis techniques. By embedding short COSI descriptions and Wikipedia article text in a shared TF–IDF vector space and quantifying relatedness via cosine similarity, we generate a continuous, real-valued relevancy matrix that captures graded differences in topical alignment. Despite the limited size of the COSI reference texts, the approach identifies interpretable domain-specific vocabulary and reveals meaningful variation in how computational biology topics are represented across open educational resources.

TF–IDF provides an established statistical framework for identifying terms that are salient within a document while remaining discriminative across a collection (Salton and Buckley 1988). TF-IDF has proven effective in a variety of contexts; in this study, we illustrate its use in the context of educational resources in computational biology. TF–IDF enables the identification of vocabulary that is characteristic of specific ISCB COSIs, while down-weighting terms that are common across computational biology more broadly. This distinction is essential in a field where general methodological terminology coexists with highly specialized language that signals domain relevance. Applying TF–IDF to systematically preprocessed COSI descriptions and educational resource texts enables the extraction of domain-specific lexical features. This representation captures not only individual technical terms but also compound expressions that encode conceptual or methodological specificity. The resulting feature vectors provide a quantitative basis for similarity analysis, enabling educational resources to be compared against COSI descriptions in a principled and reproducible manner. Using the ISCB COSIs as a reference corpus grounds relevance assessment in established community structures rather than externally imposed taxonomies. This approach supports the quantitative evaluation of topical coverage across educational resources, facilitates the identification of gaps or redundancies within subdomains, and illustrates how lightweight natural language processing methods can be used to operationalize community-defined knowledge structures in computational biology (Manning *et al.* 2008).

In this study, we used TF-IDF in combination with cosine similarity as a statistical metric. TF-IDF is particularly strong in terms of transparency and interpretability (Ayash *et al.* 2025); however, TF-IDF may underperform when semantically related texts use different terminology. More modern context-aware NLP methods, such as semantic embedding or transformer-based language models, may provide more effective quantification of domain relevance for highly specialized articles where lexical overlap with COSI descriptions is limited. Exploring these techniques represents a promising direction for future work.

The relevancy matrix presented in this study extends the original binary relevancy matrix and further allows COSIs to rank Wikipedia articles by topical relevance to their domain, an advantage over the previous COSI-Article matrix (Kilpatrick *et al.* 2022). Unsupervised analysis of the relevancy matrix further reveals coherent structure within the data. Dimensionality reduction using principal component analysis followed by UMAP projection preserves neighbourhood relationships between articles, enabling visual exploration of topical similarity. Construction of shared nearest-neighbour graphs and application of Leiden community detection identifies clusters of Wikipedia articles that correspond to specific COSIs or groups of closely related COSIs. These clusters recover known conceptual relationships within computational biology, such as links between machine learning, systems modelling, and structural bioinformatics, while also highlighting subdomains that remain weakly represented or diffuse across the article corpus. The concordance between cosine similarity–based analyses and graph-based clustering supports the internal consistency and robustness of the framework.

The binary scoring of the previous COSI-Article matrix also had inherent limitations in its assumptions of equal relevancy. For example, Wikipedia articles classed as relevant to multiple COSIs were assumed to also be equally relevant to those COSIs. While this may be true in some cases, we suggest that more often than not, articles are more or less relevant to one COSI than another. Similarly, there was an assumption that all articles marked as relevant to a single COSI were equally relevant. Again, we suggest that this is not the case, and that extending relevancy scores to a real number better reflects the true relevancy of articles to given COSIs. While TF-IDF produces a continuous relevance score, practical applications may still require thresholds to identify subsets of articles (e.g. for recommended reading lists). Such thresholds would likely vary between COSIs, given the different score distributions observed above. Consequently, percentile-based or rank-based thresholds may be more appropriate than a fixed threshold for these applications.

A further advantage of the TF-IDF method presented here is the unbiased nature of the relevancy scoring, based only on summary text for each COSI and the current main text from each Wikipedia article. For example, in our previous study, volunteers identified the Education COSI as having the highest number of relevant articles (Kilpatrick *et al.* 2022). We observed that many biographical articles in particular were associated with the Education COSI, with the motivation for that classification based on the subject’s role as an educator, but not necessarily relating to bioinformatics pedagogy. We also noted an argument that all Wikipedia articles are educational to some extent, but again not relating to pedagogy. The scoring method presented here removes such arguments and unconscious bias by scoring relevance based solely on the provided text. While a quantitative assessment of the reduction in bias afforded by TF-IDF was not feasible for this study, future work could compare article relevance rankings (e.g. by correlating with human-derived article rankings) to formally quantify this shift.

Our analysis suggests that despite the small amount of text used to summarize COSI activity in the TF-IDF input, some COSI-specific terms are identified. More expansive text inputs may be generated from ISMB proceedings abstracts for each COSI. These inputs would include more complex jargon and compound expressions typical of each COSI, in comparison with the short COSI descriptions used in this study. While we acknowledge a human element in assigning proceedings abstracts to COSIs, we speculate that such an approach may provide even better results. Similarly, we acknowledge that very short or highly specialized Wikipedia articles may receive unreliable relevancy scores due to limited lexical overlap with COSI descriptions, even when they are conceptually related to a given domain; the YeTFaSCo article described above is an example of this, with a score of 0 for the BOKR (Bio-Ontologies and Knowledge Representation) COSI.

Currently, Wikipedia editors identify and tag articles as relevant to computational biology. We have previously suggested that this is another area for potential editor subjectivity: human editors may miss relevant articles, or either consciously or unconsciously decide against tagging articles as relevant to computational biology (Kilpatrick *et al.* 2022). This issue may be exacerbated by editors with no knowledge of computational biology. The scalability of the automated system presented here suggests a clear opportunity for future work to reduce this human subjectivity, by systematically identifying (and/or tagging) existing articles from the full Wikipedia corpus (as of January 2026, over 7.1 million) that are relevant to one or more COSIs but not currently tagged as being relevant to computational biology. The current system allows for identification of such articles, but could be extended to actually carrying out the tagging procedure on Wikipedia, either via Wikipedia’s API or the Pywikibot library, a framework for creating Wikipedia bots to perform maintenance tasks (Pfundner *et al.* 2015).

The method for identifying articles in other languages will work effectively across languages, including Spanish, provided that language-specific preprocessing steps such as selecting appropriate stop points and term normalization are applied. There is no inherent limitation in the approach that restricts it to English; rather, successful cross-language application depends on adapting tokenization and stop lists to the target language. This adaptation can leverage the language modelling and multilingual knowledge graph strategies outlined by Sélem-Mojica *et al.* (2024, 2025), which demonstrate robust performance in multilingual contexts and provide a framework for extending the method to additional languages (Sélem-Mojica *et al.* 2024, 2025).

## 5 Conclusion

This study highlights that although the ISCB COSI system was organic in the way the communities were formed and topics included under their umbrella were organized, through the use of textual analysis we were able to regenerate many of the local networks of topics within these groups. We note the framework used for our case study on the ISCB COSIs is unsupervised and can be used on larger article corpuses. By systematically identifying Wikipedia articles relevant to volunteers with domain-specific expertise, this classification method can ensure even small communities can improve articles within their domain that may be overlooked, improving a vital open educational resource in computational biology.

## Data Availability

The relevance matrix developed in this study is available in the Zenodo repository (doi: 10.5281/zenodo.18311878).
